# Internet of Things-Based Wayfinding for Hospital Visitors: A Digital Solution for Complex Health Care Infrastructures

**DOI:** 10.1016/j.mcpdig.2025.100293

**Published:** 2025-10-08

**Authors:** Prajwal L. Salins, Ganesh Anandan, Basilio Duke Ananda, Bhageerathy Reshmi, Roshan David Jathanna

**Affiliations:** a Department of Health Information Management, Manipal College of Health Professions, Manipal Academy of Higher Education, Manipal, Karnataka, India; b Manipal Institute of Technology, Manipal Academy of Higher Education, Manipal, Karnataka, India

## Abstract

**Objective:**

To design, implement, and evaluate a digital indoor wayfinding web application (KH Wayfinder) for a tertiary care hospital, assessing its effects on spatial orientation and navigation-related stress among visitors.

**Participants and Methods:**

A 3-phase study was conducted in a tertiary care hospital in coastal Karnataka, India, from April 1, 2023, through July 31, 2024. Phase 1 involved a cross-sectional survey (n=41) to assess user attitudes toward digital wayfinding. In phase 2, a browser-based application was developed using HyperText Markup Language, JavaScript, cascading style sheets, and Leaflet.js, covering 5 hospital floors with 52 destination points and 758 routes. Phase 3 consisted of usability testing with 54 participants using a validated questionnaire to assess performance, satisfaction, and ease of use.

**Results:**

The majority of users 33 (80.5%) expressed willingness to use a digital Wayfinder. Postimplementation results showed that 46 (85.2%) found the tool easy to use, 47 (87%) reported a reduction in navigation time, and 45 (83.3%) experienced reduced psychological stress. Additionally, 51 (94.4%) preferred the digital system over traditional signage, and 54 (100%) would recommend it to others.

**Conclusion:**

KH Wayfinder demonstrated high usability, effectiveness, and user satisfaction as a low-cost digital navigation solution. Its browser-based architecture and open-source design make it scalable and adaptable for broader use in smart hospital environments. Future enhancements may include real-time positioning, multilingual support, and accessibility features.

Spatial complexity is increasingly challenging in contemporary health settings, especially in tertiary care settings.^1^ There has been an increase in the size and function of hospitals to cater to specialized services and increased patient admissions. Thus, wayfinding presents itself as the next operational and experiential challenge.^2^ Visitors and patients often struggle to locate clinics, diagnostic areas, or administrative counters within sprawling hospital campuses.^3^ Studies show that up to 30% of hospital visitors experience disorientation or anxiety while navigating to unfamiliar departments, leading to missed appointments, delayed consultations, and increased demand on frontline staff for nonclinical support.^4^ This disorientation ultimately affects patient satisfaction and the efficacy of workflow, as staff find themselves spending time helping patients navigate the building.^5^^,^^6^

Internet of Thing (IoT)-enabled digital wayfinding systems have come forward as a promising alternative.^7^^,^^8^ Such systems capitalize on interactive technologies such as web-based applications, real-time data, and geolocation services to navigate steps and pleasingly enhance the user-friendly ambience in hospital environments.^9^^,^^10^ With digital signage, the controls of the digital system, such as direction, can vary depending upon user inputs.^11^ It can offer step-by-step directions to clients that can be altered when the infrastructure or the service locations change down the road. Most importantly, these systems allow the user to be pretty much self-dependant, thereby reducing staff support interruptions caused to clinical workflows. A 2021 study by Ženka et al^12^ found that over 60% of hospital users under age 40 years prefer smartphone-based navigation, whereas older or less digitally literate users still rely on human assistance. Furthermore, 87.5% of respondents in the same study indicated that digital navigation tools helped reduce stress and improve wayfinding accuracy in hospital environments.^12^^,^^13^

This study presents the design, development, and evaluation of an IoT-based digital indoor wayfinding system implemented in a tertiary care hospital in coastal Karnataka, India. The project was executed in phases—initially developing a web-based prototype for the outpatient department (OPD) building and then improving it based on user feedback and usability testing. The system combined interactive maps, direction animations, and a mobile-responsive user interface, developed with common web technology stacks such as HyperText Markup Language (HTML), JavaScript, cascading style sheets (CSS), and Leaflet.js. Providing an avenue between a static navigation tool and a user-centric digital version, the research adds to the increasing evidence toward IoT-based applications within hospitals. The conclusions advocate for a reduction in cognitive and physical strain caused by hospital navigation with the system set up; later enhancements would include multilingual support, voice-assisted routing, and linkage with hospital appointment systems. As health care institutions continue to prioritize patient-centric service models, digital wayfinding stands as a critical enabler of improved access, efficiency, and experience.

## Participants and Methods

This study was conducted in 3 distinct phases to systematically design, develop, and evaluate a digital indoor wayfinding solution for a tertiary care hospital in coastal Karnataka, India, from April 1, 2023, through July 31, 2024. The overall methodological framework combined user-centered design principles, waterfall software development methodology, and both quantitative and qualitative evaluation techniques to ensure technical robustness and practical feasibility.

### Phase 1: Cross-Sectional Attitude Assessment

To understand the navigational challenges faced by hospital users and assess the perceived need for a digital wayfinding solution, a cross-sectional study was conducted among patients, visitors, hospital staff, and students. Using purposive sampling, a validated questionnaire was administered to 41 persons aged 18 years and above, all of whom owned smartphones with internet access. Those with visual impairment or illiterate were excluded. The questionnaire collected information on familiarity with indoor navigation tools, hospital visits frequency, most preferred means of asking directions, and willingness to use a digital-based wayfinding solution. The tool was validated through principal component analysis and was used to extract insights on user attitudes and expectations.

### Phase 2: Application Development Using Waterfall Methodology

**Figure 1:**
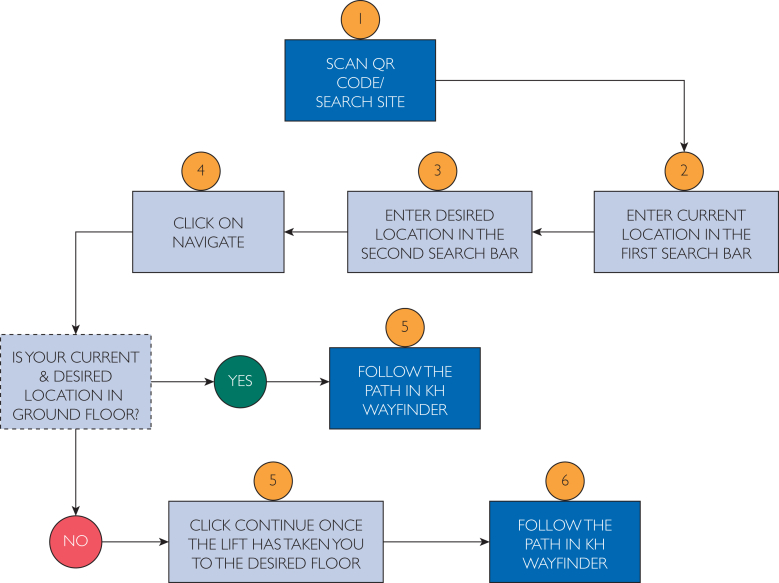
User navigation flow in the KH Wayfinder application. The flowchart illustrates the sequential steps for using the system: scanning a QR code or accessing the search site, entering the current and desired locations, clicking on the “Navigate” option, and following the indicated route. If the destination is on the ground floor, users directly follow the displayed path; if not, they are prompted to use the lift and continue navigation on the appropriate floor.

The digital indoor wayfinding system was developed using the waterfall software development model, which is particularly suitable for structured projects with clearly defined phases. A total of 52 major locations and 758 navigation paths were considered and plotted accordingly. Transparent, traceable, and modular development followed the 5 major steps: requirement gathering, system designing, implementation, testing, and deployment. The system is a light web application that runs on most smartphone browsers and desktop computers without having to install a native application, allowing the hospital administration and visitors some flexibility. The user navigation flow in the KH Wayfinder application is illustrated in Figure 1.

#### Requirements Analysis and Floor Plan Integration

The process began with a thorough analysis of user needs, extracted from stakeholder discussions and survey data (from phase 1), as well as physical walkthroughs of the hospital premises. The choice for initial intervention was the OPD because it records heavy patient traffic and is difficult to navigate. For input into the map, raw data consisted of printed architectural floor plans of the existing and new OPD buildings spanning 5 floors, which were scanned and amalgamated in Adobe Illustrator to create a composite vector map for each floor. Key locations, consultation rooms, diagnostic areas, billing counters, help desks, and elevators were geotagged in relation to each other using spatial estimation techniques and further verified by on-ground coordinates. A total of 52 destination nodes and 758 navigable path segments were geotagged to form distributed graph structures spanning the 5 floors of the hospital.

#### Technical Stack and System Architecture

The frontend of the system was developed using HTML5, CSS3, and Vanilla JavaScript for the unparalleled browser support and speedy development processes. The interactive mapping and routing engine was built in Leaflet.js, an open-source JavaScript library for mobile-friendly interactive maps. Leaflet is capable of supporting custom tile layers while rendering large-scale vector graphics efficiently on desktop and mobile. Each floor map was cast as a custom raster layer, georeferenced, and imposed on the Leaflet map canvas with .setBounds() to have positional integrity across all floor levels. Custom marker icons were set up for source and destination points. The route animation was done using the Leaflet-AntPath plugin that animates a dashed polyline from one point to another, thus cementing spatial orientation in the mind of the user. Green and red markers were used to mark starting and ending points, with optional destination labels to reinforce comprehension. Dropdown menus were implemented using standard HTML select elements, populated dynamically with JavaScript arrays linked to the internal map graph. When a user selects a source and destination pair, the application retrieves the associated path from a predefined path matrix (stored as a JavaScript object or JSON file) and dynamically draws the animated route on the canvas.

#### Location Mapping and Way Graph Creation

A manual spatial pathing system was implemented due to the absence of real-time location tracking in the initial version. Each route between any source and destination was prequalified or had its name stored as a set of ordered coordinate pairs. The paths were constructed using a very lightweight internal graph model, wherein each destination point acted as a node and the hallways or corridors that could be walked along were edges. During implementation, the navigable paths were created manually using some kind of spatial framework tool like Leaflet.Draw, and they were stored as sequences of latitude-longitude (or pseudocoordinate) values that kept up with the resolution of the floor map; these were then assembled into a static route database from which routes could be efficiently retrieved at runtime. The application left out any form of real-time geolocation with global positioning system or Bluetooth low energy owing to the lack of infrastructure but was kept modular for the potential integration of indoor positioning systems.

#### Testing and Debugging

Following implementation, the application underwent iterative component-level testing, including the following:

• functional testing of dropdown routing logic;
• rendering checks for map boundaries and path accuracy;
• mobile responsiveness testing across browsers (eg, Chrome, Safari, and Edge) and devices (eg, Android and iOS);
• stress testing for map rendering with multiple path overlays.

Errors related to marker misalignment, incomplete paths, and dropdown synchronization were logged and resolved during this stage. The final version was fully compatible with modern browsers and touch-enabled devices.

#### Deployment

The final application was deployed using GitHub Pages, a free and static web hosting service that integrates directly with Git version control. This deployment model was chosen for its simplicity, cost-efficiency, and ease of access. The deployed application was made available publicly via a static URL (https://ganeshanandd.github.io/KH_Way_Finder/), making it accessible on any device with a browser and internet connection. This architecture ensured low maintenance overhead and zero hosting costs, making it an ideal prototype for public hospital settings where budget constraints are a concern.

### Phase 3: Usability and Feasibility Evaluation

Following development, testing for feasibility was conducted with regard to the functionality and user experience of the application as it was being deployed. Fifty-four participants from hospital publics (different from those of phase 1) were recruited using purposive sampling. The same inclusion and exclusion criteria applied. They were instructed to use the application for navigation to certain preset locations within the hospital. Upon completion of the navigation, a previously validated questionnaire was administered that focused on ease of navigation, interface clarity, reduction in navigation time, and general satisfaction. The study also gathered user suggestions for possible improvements. The feedback data were examined again using the principal component analysis approach. Key performance indicators included perceived ease of use, accuracy of routes, confidence in navigation, and reduction of stress due to hospital visiting.

## Results

The development and evaluation of the IoT-based digital indoor wayfinding system, KH Wayfinder, were carried out in 3 systematic phases: (1) a cross-sectional study to assess user attitudes and baseline expectations; (2) full-stack web application development and deployment focused on the hospital’s OPD; and (3) real-world usability testing to evaluate its effectiveness, accessibility, and potential for scale. The findings from each phase are presented further.

### User Awareness and Attitude Assessment

In phase 1, 41 participants, representing patients, visitors, students, and hospital staff, were surveyed to understand their experiences and preferences concerning indoor navigation in health care environments. The mean age of the participants was 40.8±13.3 years, indicating a broad adult age distribution. In terms of gender, 24 participants (59%) were men and 17 (41%) were women. The participants represented 4 key end-user groups: patients 20 (49%), visitors 6 (15%), students 8 (19%), and hospital staff 7 (17%). With regard to educational background, most respondents were educated to at least the undergraduate level, with 11 (27%) having completed higher secondary education, 25 (61%) holding a bachelor’s degree, and 5 (12%) a master’s degree. Moreover, 31 (75.6%) of respondents stated they had never used any sort of indoor navigation system, an observation derived despite the fact that all respondents owned smartphones. This was indicative of a technology-existence versus technology-adoption gap within the health care wayfinding system—the gap now was borne of lack of provision rather than unwillingness. When asked how they currently managed to find a place within the hospital, 23 (56.1%) said that they would ask hospital staff or strangers, whereas 16 (39%) depended on wall signage and printed directions. Only 2 (4.9%) indicated having no preference or remaining indifferent. On the contrary, despite their traditional and static means, a substantial 33 (80.5%) of participants indicated that they would adopt a digital wayfinding application should one be launched. A total of 36 (87.8%) were of the opinion that such a system would reduce confusion, help in self-navigation, and make hospital visits much less stressful. Participants also suggested a range of features they would like to see integrated into the digital navigation system. These included Quick Response code scanning for quick access to navigation routes 18 (43.9%), multilingual support 5 (12.2%), voice guidance 2 (4.9%), real-time location updates 3 (7.3%), and integration with mHealth platforms 1 (2.4%). These findings informed both the design specifications and future enhancement roadmap of the application.

### System Deployment and Feature Implementation

**Figure 2:**
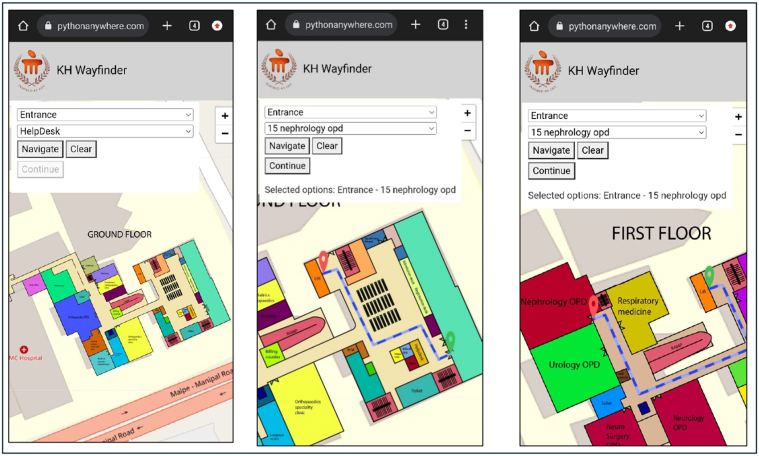
User interface and routing visualization of the KH Wayfinder application across hospital floors. Screenshots show the navigation process from entrance to nephrology outpatient department (OPD), including the selection of source and destination points and the display of routes on both the ground and first floors.

In phase 2, the KH Wayfinder-P was developed using a collection of lightweight yet open-source web technologies to achieve maximum compatibility and responsiveness along with a minimal hosting overhead. From this, it can be observed that the application used HTML5 for layout and structure, CSS3 for decorating and styling, while interaction logic was implemented using Vanilla JavaScript. Leaflet.js formed the base map engine, working toward rendering own hospital floor plans and route paths. Spatial mapping involved the digitization and merging of architectural maps for both old and new OPD buildings using Adobe Illustrator toward creating a unified and color-coded navigational interface. The entire application had mapped 5 floors of the hospital along with 52 key locations, including OPDs, diagnostics, billing counters, and reception desks. The paths-to-route combinations of sources-destinations, that is, 758 in total, were manually created and stored in an internal data structure accessible through an interface with a dropdown. The application offered the ability to animate directional lines using the AntPath plugin^14^; to mark the start and end points, custom-color markers were used, which further assisted the user. During development, iterative testing confirmed correct route rendering, marker placement accuracy, interface responsiveness across devices, and compatibility with major browsers. Special attention was given to mobile optimization because smartphones were the primary mode of access anticipated by end users. The application was ultimately deployed via GitHub Pages, offering zero-cost static hosting with public accessibility through a dedicated URL. The interface and routing visualization of the KH Wayfinder across floors is shown in Figure 2.

### Usability Testing and Feasibility Assessment

**Figure 3:**
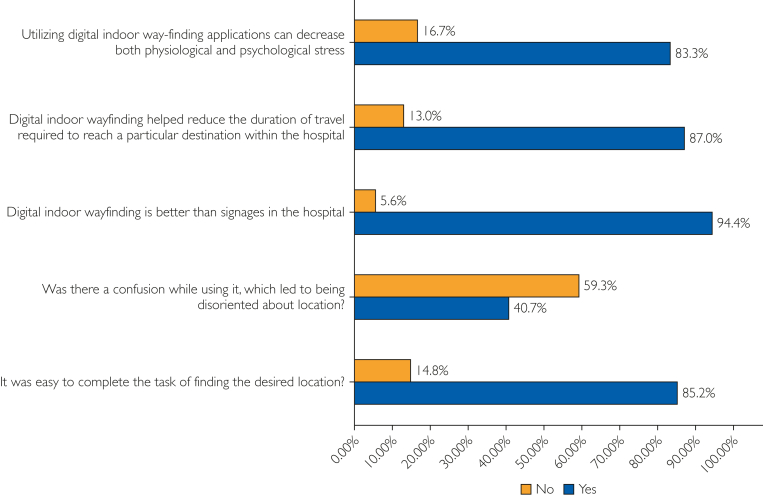
User perceptions and usability outcomes from KH Wayfinder based on phase 3 validated questionnaire (n=54). The bar chart presents participant responses on stress reduction, reduced navigation time, preference over traditional signage, instances of confusion, and overall ease of completing navigation tasks.

In phase 3, the live system was tested by 54 new participants from the hospital public. The average age was 38.2±12.6 years, showing that the cohort was slightly younger than that of phase 1. Sample included 25 males (46%) and 29 females (54%), indicating a relatively balanced distribution. The participants were again drawn from key end-user categories: patients, 18 (33%); visitors, 16 (30%); students, 4 (7%); and hospital staff, 16 (30%). Regarding education levels, most participants had attained higher education, with 9 (17%) completing higher secondary and 45 (83%) holding a bachelor’s degree. These users interacted with KH Wayfinder in real-time, using it to locate specific destinations within the hospital. They then provided feedback via a structured, validated questionnaire. The usability findings were overwhelmingly positive. User perceptions and usability outcomes from the phase 3 validated questionnaire are summarized in Figure 3. The complete instruments are available in Supplemental Appendixes 1 and 2 (available online at https://www.mcpdigitalhealth.org/).

### Ease of Use

Approximately 46 (85.2%) of participants reported that navigating to the intended destination was easy. Of these, 48 (89.1%) attributed the ease to the clarity of the map and on-screen directions. Only 8 participants (14.8%) faced difficulties, primarily due to two technical limitations: a less intuitive dropdown search interface, reported by 5 participants (62.5%) of those dissatisfied, and occasional glitches in real-time location detection, reported by 2 participants (25%).

### Reduction in Time

In total, 47 (87%) of participants self-reported that the system significantly reduced the time required to reach their desired destination. This finding aligns with previous studies in digital navigation literature, where travel efficiency is considered a core performance metric.

### Stress Reduction

Approximately 45 (83.3%) of users agreed that using the system helped reduce the psychological and physiological stress of navigating an unfamiliar hospital environment. Participants noted reduced anxiety, increased independence, and less reliance on hospital staff.

### Comparative Advantage Over Traditional Signage

A remarkable 51 (94.4%) of users stated that they preferred the digital system over conventional signage, citing clarity, convenience, and interactivity as key differentiators. Importantly, 54 (100%) of users said they would recommend KH Wayfinder to others, validating its perceived value and usability. Participants also offered actionable feedback for further enhancement. Suggestions included improvements to the dropdown search (10, 18.5%), better live location tracking (8, 14.8%), voice navigation support (9, 16.7%), map orientation features (3, 5.6%), and department timing integration (1, 1.9%).

### Qualitative Insights and Observed Behavior

Other than survey metrics, field observations during the testing period showed some noticeable behavioral trends. A 32 of 54 participants (59.3%) of users were observed by themselves gravitating toward the application interface without requiring much assistance after the task was explained to them. Users were also seen exploring various route combinations on their own, hinting at a very high level of intuitiveness. Among older users (n=9), 6 participants expressed initial hesitance to make use of the system but found themselves quite satisfied after the first time they were shown how to do it. This points to the fact that little onboarding is a real key to driving adoption, especially for the less digitally literate users. This means that if the tool is rolled out into other hospital blocks, it could bring real operational benefits by easing the burden on frontline staff, allowing them to concentrate on more important functions.

## Discussion

The implementation and evaluation of KH Wayfinder illustrate a growing need and readiness for digital indoor navigation systems in health care settings. The high levels of acceptance and usability reported by hospital visitors align with broader trends in smart health care infrastructure, where digital tools are increasingly used to enhance patient-centered care.^10^^,^^15^ This study’s experimentation with digital navigation showed that a simple, browser-based solution could do well to enhance navigation experiences within complex operating layouts. This aligns with recent work by Zhou,^16^ who highlighted how QR-integrated digital navigation systems help to improve patient flow and resolve confusion in clinical settings. Our findings lend support to the conclusion of that study, especially user-reported decreases in psychological stress and better time delivery to clinical areas.

In phase 3, 31 of the 54 participants (57.4%) were younger than 40 years. This subgroup showed a clear preference for mobile-based navigation, which aligns with prior literature describing generational and educational differences in wayfinding behavior. Our findings therefore corroborate the patterns reported by Ženka et al,^12^ who noted that younger and more digitally literate users tend to favor smartphone-based navigation, whereas older participants are more likely to rely on personal assistance. Their research revealed that users younger than 40 years preferred smartphone-based navigation, whereas older individuals and those with lower educational backgrounds favored personal assistance.^12^ Moreover, the observed decline in staff interruptions for direction-seeking corroborates the findings of the study by Majerova et al,^17^ which demonstrated tangible economic and operational benefits from indoor digital navigation deployment in hospital systems.^17^

Although prior research has explored beacon-based or global positioning system–integrated solutions for indoor navigation, many of these depend on expensive infrastructure or suffer from location accuracy issues due to indoor interference. Our approach, using predefined spatial mapping via Leaflet.js and AntPath animation, provides a scalable alternative that avoids these technological hurdles. Although the system currently lacks real-time positioning, its modular architecture allows for future integration with Bluetooth low energy, Wi-Fi RTT, or vision-based systems, which have been suggested in recent scoping reviews by Wichmann^18^ and Huang et al,^19^ as promising for indoor positioning in health care. This highlights the potential of KH Wayfinder not only as a functional standalone system but also as a platform for hybrid navigation models in the future.^18^^,^^19^

The overwhelmingly positive response to the application also reaffirms the importance of inclusive interface design in health care IT. Accessibility features such as mobile responsiveness, visual markers, and animated routing reduce entry barriers for first time users and for individuals unfamiliar with digital tools. The system gained so much traction that it suggested even a low-cost open-source application, designed around human-centered principles, can take huge strides in bettering the hospital experience. Future studies may want instead to validate such systems in different types and locations of hospitals, incorporating longitudinal outcomes including repeat visits, appointment adherence, and further layouts optimized to staff workflow.

## Conclusion

The KH Wayfinder system successfully addressed the navigation challenges faced by hospital visitors through a cost-effective, browser-based digital solution. User satisfaction and acceptance levels had been very high, proving it as a beneficial digital intervention in such a complex health care environment. KH Wayfinder thus fills a vital usability gap and lays down a flexible foundation for future extensions such as real-time localization, multilingual support, and accessibility for visually impaired users. This successful intervention endorses the growing trend toward a smart hospital ecosystem where patient experience can be enhanced by the implementation of digital navigation systems, thereby reducing staff burden and supporting operational efficiency. As hospitals are becoming more and more interconnected, wayfinding tied to electronic health record, appointment scheduling, and health information system would have its impact multiplied.

## Potential Competing Interests

The authors report no competing interests.

## Ethics Statement

The institutional ethical committee permission (IEC:194/2023) and departmental administrative permission were obtained prior to the conduct the research study. The ethical guidelines of the 1975 Declaration of Helsinki was followed. All patients in the study signed a written informed consent before participation. The physical records are stored at the Manipal College of Health Professions, Manipal Academy of Higher Education, Manipal.
