# Supplementary material for: Internet of Things-Based Wayfinding for Hospital Visitors: A Digital Solution for Complex Health Care Infrastructures
Source: Mayo Clin Proc Digit Health. 2025 Oct 8;3(4):100293. doi: 10.1016/j.mcpdig.2025.100293 (PMC12613019; doi:10.1016/j.mcpdig.2025.100293)
Supplement: Supplementary Material [file mmc1.docx]

**Supplemental Material**

**Supplementary File 1:** Scale to assess the knowledge towards the digital indoor wayfinding applications among the hospital public in the hospital.

**Sociodemographic data**

1. Gender:
2. Age:
3. Education level: High school/ higher secondary / bachelor’s degree/ master’s degree/ doctoral degree
4. Participants: patient/ visitor/ staff/ student
5. Are you aware of the Digital indoor wayfinding applications?
   1. Yes
   2. No
6. Do you use any smartphone device?
7. Yes
8. No
9. Have you ever used any digital indoor navigation system in a hospital or healthcare facility?
10. Yes
11. No
12. How often do you visit hospitals or healthcare facilities?
    1. Regularly
    2. Occasionally
    3. Rarely
13. When seeking directions inside a hospital, what kind of assistance do you prefer?
14. Asking hospital staff or volunteers/Random person
15. Using a mobile app or device for guidance
16. Relying on printed maps and signs
17. I have no preference
18. If given the option, would you be interested in using a digital indoor navigation system to navigate healthcare facilities?
19. Yes, I'm interested
20. Maybe, I'd like to learn more
21. No, I prefer other methods
22. Digital indoor wayfinding applications are helpful in navigating complex indoor spaces.
    1. Agree
    2. Disagree
23. If you were to suggest new features to this Digital indoor wayfinding application, what would be some important features that you would look for?

**Supplementary File 2:** Scale to assess the feasibility of the developed digital indoor wayfinding system among the hospital public.

**Sociodemographic data**

- 1. Gender:
  2. Age:
  3. Education level: High school/ Higher secondary / bachelor’s degree/ master’s degree/ doctoral degree
  4. Participants: patient/ visitor/ staff/ student

1. How was the experience with our Digital wayfinding website on your smartphone?

1. Good
2. Neutral
3. Bad

2. It was easy to complete the task of finding the desired location.

1. Agree
2. Disagree

Reason:

3. There was confusion while using it, which led to being disoriented about location.

1. Agree
2. Disagree

If you agree, what was the confusion:

4. Digital indoor wayfinding is better than signages in the hospital.

1. Agree
2. Disagree

5. Digital indoor wayfinding helped reduce the duration of travel required to reach a particular destination within the hospital.

1. Agree
2. Disagree

6. Utilizing Digital indoor wayfinding applications can decrease both physiological and psychological stress.

- 1. Agree
  2. Disagree
  3. Neutral

7. How likely are you going to recommend digital wayfinding over hospital signage to others?

1. Likely
2. Not likely

8. If you were to suggest new features to this Digital indoor wayfinding application, what would be some important features that you would look for?
